# Analysis of Risk Factors for Major Adverse Cardiovascular Events in Patients with Coronary Stent Restenosis after Revascularization

**DOI:** 10.31083/j.rcm2405146

**Published:** 2023-05-18

**Authors:** Zhuoxuan Yang, Tianjie Wang, Ying Dong, Long Liu, Xuan Xue, Jine Wu, Liuyi Hao, Jiansong Yuan, Jingang Cui, Shubin Qiao, Weixian Yang

**Affiliations:** ^1^Department of Cardiology, Yuncheng Central Hospital of Shanxi Province, 044000 Yuncheng, Shanxi, China; ^2^Department of Cardiology, State Key Laboratory of Cardiovascular Disease, Fuwai Hospital, National Center for Cardiovascular Diseases, Chinese Academy of Medical Sciences and Peking Union Medical College, 100037 Beijing, China; ^3^Department of Graduate, Changzhi Medical College, 046012 Changzhi, Shanxi, China

**Keywords:** coronary in-stent restenosis (ISR), myocardial infarction, target vessel revascularization, triiodothyronine (FT3)

## Abstract

**Background:**

To investigate the risk factors for myocardial infarction, recurrent in-stent restenosis (ISR) and target vessel revascularization (TVR) in patients with coronary ISR within 4 years after revascularization.

**Methods:**

A total of 1884 patients who were hospitalized at Fuwai Hospital for ISR and successfully treated with coronary intervention between January 2017 and December 2018 were included to determine whether there were myocardial infarction, recurrent ISR, TVR and other major adverse cardiovascular events (MACEs) within 4 years after intervention. The patients were divided into the MACE group (215 patients) and the non-MACE group (1669 patients). The clinical data of patients in the two groups were compared, and the risk factors for postoperative MACEs in the ISR patients were obtained by multivariate logistic regression analysis. The receiver operating characteristic (ROC) curve was used to determine the optimal prediction threshold for postoperative MACEs in ISR patients. The difference in survival curves between the two groups was compared using Kaplan‒Meier survival analysis.

**Results:**

The albumin (43.42 ± 4.77 vs. 44.17 ± 4.46, *p* = 0.021), direct bilirubin (2.5 (2, 3.5) vs. 2.8 (2.07, 3.73), *p* = 0.036) and free triiodothyronine (FT3) (2.85 ± 0.43 vs. 2.92 ± 0.42, *p* = 0.019) levels in the MACE group were significantly lower than those in the non-MACE group, and there was a significant negative correlation between albumin and FT3 and MACEs. The results of univariate and multivariate logistic regression analyses revealed that FT3 was an independent predictor of postoperative MACEs in ISR patients (Odds Ratio (OR) = 0.626, 95% CI: 0.429–0.913, *p* = 0.015). The ROC curve analysis determined that an FT3 value of 2.785 pmol/L was the optimal prediction threshold. According to the threshold, ISR patients were divided into the FT3 <2.785 group and the FT3 ≥2.785 group. The Kaplan‒Meier analysis revealed that the postoperative recurrence rate of MACEs of the FT3 <2.785 group was substantially greater than that of the FT3 ≥2.785 group (Hazard Ratio (HR) = 0.76, 95% CI: 0.58–0.994,* p *= 0.044).

**Conclusions:**

FT3 can be used as an independent predictor of postoperative myocardial infarction, recurrent ISR and TVR in ISR patients. When FT3 is <2.785 pmol/L, the incidence of postoperative myocardial infarction, recurrent ISR and TVR in ISR patients increases significantly.

## 1. Introduction

The prevalence of in stent restenosis (ISR) after bare-metal stent implantation is approximately 16% to 44%, but with the widespread use of drug-eluting stents, the incidence of ISR decreases to 5% to 15% [1]. However, the number of patients receiving coronary stent implantation is increasing yearly. In 2021, the total number of coronary interventional procedures in mainland China exceeded 1.16 million, with an average of 1.48 stents or drug-coated balloons per patient [2]. As a result, the total number of patients with ISR remains negligible. Some studies have suggested that the pathogenesis of ISR is due to the damage to blood vessels that is caused by stent implantation, which triggers a series of local and systemic chain reactions. The final result of these reactions determines whether the vascular endothelium forms a smooth and thin intima or stent restenosis occurs [3]. This pathological process includes the activation of endothelial cells after injury, platelet degranulation and aggregation [4, 5], the release of growth factors and cytokines, the proliferation and migration of smooth muscle cells (SMCs), an increase in extracellular matrix synthesis [3], bone marrow endothelial progenitor cells [6, 7, 8], and inflammatory responses.

After receiving revascularization therapy, some of these patients still had short-term or long-term major adverse cardiovascular events (MACEs), such as myocardial infarction, recurrent ISR (reISR) and target vessel revascularization (TVR). This not only imposes a huge financial burden on patients and their families but also imposes a severe psychological burden. Currently, there are few studies of MACEs in patients with ISR after revascularization, and there are no accepted predictors. The aim of this study was to investigate predictors of myocardial infarction, recurrent ISR and TVR in patients with ISR following revascularization intervention.

## 2. Subjects and Methods

### 2.1 Study Population 

A retrospective analysis was conducted that included 1884 patients, 1494 males and 390 females, who were admitted to Fuwai Hospital for ISR and successfully received coronary intervention between January 2017 and December 2018. ​According to the presence or absence of myocardial infarction, recurrent ISR and target vessel revascularization during the follow-up, the patients were divided into a MACE group (n = 215) and a non-MACE group (n = 1669). The median follow-up duration was 35 months.

Each individual signed an informed consent form. The Fuwai Hospital’s ethical committee approved this study.

The inclusion criteria were as follows: (1) individuals whose ISR was verified by coronary angiography (ISR was defined as the loss of ≥50% of the coronary lumen in the area of a previously stented lesion, and stenosis within 5 mm of the edge of the stent by coronary angiography was also defined as ISR); and (2) successful revascularization.

The exclusion criteria as follows: (1) patients who had bare metal stents implanted; (2) patients with stent implantation in bypass vessels; and (3) patients with severe heart, liver, renal insufficiency, abnormal thyroid function, anemia, infection or tumor.

### 2.2 Data Collection

(1) Age, sex, height, weight, previous medical history (hypertension, diabetes, hyperlipidemia, stroke history, chronic renal insufficiency history) and other data were collected. (2) Operation information, including ISR location, number and intervention treatment (stent reimplantation or drug-coated balloon use) were collected. (3) Fasting venous blood was collected from all patients in the morning before operation, and tests for routine blood, liver and kidney functions, electrolytes, fasting blood glucose, blood lipids and thyroid function (chemiluminescent immunoassay, reference range: thyroid stimulating hormone (TSH): 0.56–5.91 μIU/mL, free triiodothyronine (FT3): 3.53–7.37 pmol/L, free thyroxin (FT4): 7.98–16.02 pmol/L), N-terminal pro brain natriuretic peptide (NT-proBNP), and other related tests were performed. The ventricular ejection fraction was determined by echocardiography, and the estimated glomerularfiltrationrate (eGFR) values were calculated using the Cockcroft-Gault formula. (4) The patients were followed up for MACEs, including occurrence time, and outpatient drug use.

### 2.3 Statistical Analysis

SPSS 25.0 (IBM Corp., Armonk, IL, USA) and R version 4.4.0 (R Core Team, 2013, Vienna, Austria) were used for statistical analyses of the data. Continuous variables are expressed as the mean ± standard deviation (normal distribution) and median (nonnormal distribution). Two independent sample *t* tests (normal distribution) or nonparametric tests were used to compare the two groups (nonnormal distribution). For comparisons between the two groups, the chi-square test was employed to describe count data as a frequency (example) and rate (%). The correlation between other variables and MACEs was described using Spearman’s correlation analysis. Univariate and multivariate logistic regression analyses (backward LR) were used to determine independent predictors of MACEs in ISR patients. Receiver operating characteristic (ROC) curve analysis was performed using R version 4.4.0 to determine the optimal threshold as well as the sensitivity and specificity of the threshold. Based on the ideal threshold, patients were separated into two groups, and survival differences between the two groups were examined using Kaplan‒Meier survival analysis. *p *< 0.05 indicated a statistically significant difference.

## 3. Results

### 3.1 Study Participants and Baseline Characteristics

There were no differences in age, sex, body mass index (BMI), history of hypertension, hyperlipidemia, stroke, diabetes, history of chronic renal insufficiency, ISR reference vessel size, ISR events, or therapy between the two groups. The percentage of patients receiving ticagrelor as an outpatient treatment in the MACE group was considerably greater than that in the non-MACE group (78 (36.3%) vs. 451 (27%), *p* = 0.006). Albumin (43.42 ± 4.77 vs. 44.17 ± 4.46, *p* = 0.021), direct bilirubin (2.5 (2, 3.5) vs. 2.8 (2.07, 3.73),* p* = 0.036) and FT3 (2.85 ± 0.43 vs. 2.92 ± 0.42, *p* = 0.019) levels in the MACE group were significantly lower than those in the non-MACE group, and there was no significant difference in other indicators between the two groups (Table 1).

**Table 1.** **Baseline characteristics of the study participants**.

		MACEs group	Non-MACEs group	*p* values
		(n = 215)	(n =1669)	
Gender (male, %)		162 (75.3%)	1332 (79.8%)	0.129
Age		60 (54, 67)	62 (55, 68)	0.081
BMI		25.35 (23.92, 27.76)	25.95 (23.94, 28.08)	0.999
History of smoking		131 (60.9%)	1055 (63.2%)	0.548
History of hypertension (%)		145 (67.8%)	1126 (67.6%)	1.000
History of diabetes mellitus (%)		92 (42.8%)	735 (44.0%)	0770
Hyperlipidemia (%)		200 (93%)	1562 (93.6%)	0.768
History of stroke (%)		27 (12.6%)	206 (12.3%)	0.912
CKD history (%)		3 (1.4%)	27 (1.6%)	1.000
Out-patient medication				
	Aspirin	207 (97.6%)	1626 (98.1%)	0.594
Adenosine diphosphate (ADP) inhibitor				0.006
	Clopidogrel	137 (63.7%)	1218 (73%)	
	Ticagrelor	78 (36.3%)	451 (27%)	
Beta receptor blocker		169 (78.6%)	1322 (79.2%)	0.858
	Angiotensin converting enzyme inhibitor /Angiotensin receptor blocker	117 (54.4%)	922 (55.2%)	0.827
	statin	215 (100%)	1669 (100%)	1.000
Number of ISR vessels				0.194
	1	189 (87.9%)	1518 (91.0%)	
	2	23 (10.7%)	134 (8.0%)	
	3	3 (1.4%)	17 (1.0%)	
ISR location				0.471
	Left anterior descending (LAD)	95 (44.2%)	785 (47%)	
	Left circumflex artery (LCX)	27 (12.6%)	222 (13.3%)	
	Right coronary artery (RCA)	73 (34%)	561 (33.6%)	
	LAD + LCX	10 (4.7%)	46 (2.8%)	
	LAD + RCA	5 (2.3%)	28 (1.7%)	
	LCX + RCA	4 (1.9%)	12 (0.7%)	
	LAD + LCX + RCA	0 (0%)	2 (0.1%)	
Treatment				0.325
	Drug Eluting Stent (DES)	114 (53%)	970 (58.1%)	
	Drug Coated Balloon (DCB)	101 (47%)	699 (41.9%)	
Left ventricular ejection fraction		62% (60%, 66%)	62% (60%, 65%)	0.549
Big endothelin		0.22 (0.16, 0.31)	0.24 (0.18, 0.34)	0.109
Preoperative brain natriuretic peptide		116.6 (45.7, 195.8)	104.2 (49.8, 244)	0.508
Glycosylated hemoglobin		6.54 ± 1.21	6.54 ± 1.18	0.562
Albumin		43.42 ± 4.77	44.17 ± 4.46	0.021
Glutamic-pyruvic transaminase (ALT)		23 (14, 35)	23 (17, 34)	0.400
	Glutamic oxalacetic transaminase (AST)	23 (17, 26)	23 (19, 28)	0.405
	Total bilirubin	12.03 (8.92, 15.4)	12.97 (10.33, 16.29)	0.423
	Direct bilirubin	2.5 (2, 3.5)	2.8 (2.07, 3.73)	0.036
	Creatinine	73.66 (66.68, 87.09)	83.54 (72.77, 94.03)	0.060
	eGFR	94.752(76.82, 117.49)	86.03 (69.95, 103.41)	0.092
	Urea nitrogen	5.31 (4.1, 6.3)	5.4 (4.4, 6.5)	0.623
	Uric acid	284.61 (242.17, 367.82)	350.2 (294.6, 407.11)	0.057
	Triglyceride	1.31 (1.09, 2.48)	1.45 (1.09, 1.96)	0.897
	Total cholesterol	3.71 (3.16, 4.69)	3.69 (3.21, 4.39)	0.874
	High density lipoprotein cholesterol (HDL-c)	1.14 (0.96, 1.39)	1.06 (0.9, 1.23)	0.666
	Low density lipoprotein cholesterol (LDL-C)	2 (1.63, 2.95)	2.12 (1.71, 2.74)	0.735
	lipoprotein(a) (Lpa)	107.2 (46.6, 306)	199 (69.52, 469.39)	0.235
	Apolipoprotein A1 (ApoA1)	1.32 (1.19, 1.66)	1.32 (1.18, 1.5)	0.890
	Apolipoprotein B (ApoB)	0.68 (0.59, 0.92)	0.71 (0.59, 0.85)	0.977
	Highly sensitive C-reactive protein (hsCRP)	1.27 (0.48, 2.52)	1.33 (0.6, 2.85)	0.527
	Erythrocyte Sedimentation Rate	7 (3, 12)	7 (3, 12)	0.689
	FT3	2.85 ± 0.43	2.92 ± 0.42	0.019
	FT4	1.15 ± 0.17	1.15 ± 0.17	0.648
	Triiodothyronine (T3)	1.04 ± 0.19	1.07 ± 0.20	0.052
	Thyroxin (T4)	7.80 ± 1.82	7.84 ± 1.64	0.437
	TSH	1.7 (0.97, 2.69)	1.7 (1.1, 2.55)	0.594
	White blood cell	6.74 ± 1.79	6.58 ± 1.63	0.300
	Neutrophil	4.35 ± 1.47	4.20 ± 1.30	0.272
	Percentage of neutrophils	63.92 ± 8.20	63.38 ± 8.04	0.535
	haemoglobin	142.93 ± 17.83	145.46 ± 15.68	0.060
	Platelet	224.08 ± 71.26	221.18 ± 58.82	0.591

CKD, Chronic Kidney Disease; MACEs, major adverse cardiovascular events; BMI, body mass index; ISR, in stent restenosis; reISR, recurrent ISR; eGFR, estimated glomerular filtration rate; FT3, free triiodothyronine; FT4, free thyroxin; TSH, thyroid stimulating hormone.

### 3.2 Analysis of Correlation

Albumin (r = –0.053, *p* = 0.021) and FT3 (r = –0.054, *p* = 0.019) were substantially adversely linked with MACEs, according to Spearman correlation analysis. There was no significant correlation between direct bilirubin and MACEs (r = –0.037, *p* = 0.109) (Table 2).

**Table 2.** **Spearmans correlation analysis**.

Variable	Index of correlation	*p*
FT3	0.054	0.019
Direct bilirubin	0.037	0.109
Albumin protein	0.053	0.021

FT3, free triiodothyronine.

### 3.3 Univariate and Multivariate Logistic Regression Analysis

The correlation indicators described above were included in a univariate logistic regression analysis, which showed that both albumin and FT3 were predictors of MACEs. Multivariate logistic regression analysis showed that FT3 was an independent predictor of postoperative MACEs in ISR patients (Odds Ratio (OR) = 0.626, 95% CI [0.429–0.913], *p* = 0.015) (Table 3).

**Table 3.** **Univariate and multivariate logistic regression analysis**.

Variable	Univariate		Multivariate	
	OR (95% CI)	*p*	OR (95% CI)	*p*
FT3	0.627 (0.43–0.915)	0.616	0.626 (0.429–0.913)	0.015
Albumin	0.964 (0.934–0.994)	0.021	0.972 (0.941–1.004)	0.085

FT3, free triiodothyronine; OR, odds ratio.

### 3.4 Analysis of Receiver Operating Characteristic (ROC) Curve and Kaplan‒Meier Survival Analysis

The ROC curve of MACEs was analyzed for FT3, and the optimal threshold for FT3 was determined using the Youden index. When FT3 <2.785 pmol/L, the sensitivity was 52.6%, the specificity was 42.8%, and the area under the curve was 54.7% (*p* = 0.025).

**Fig. 1.**
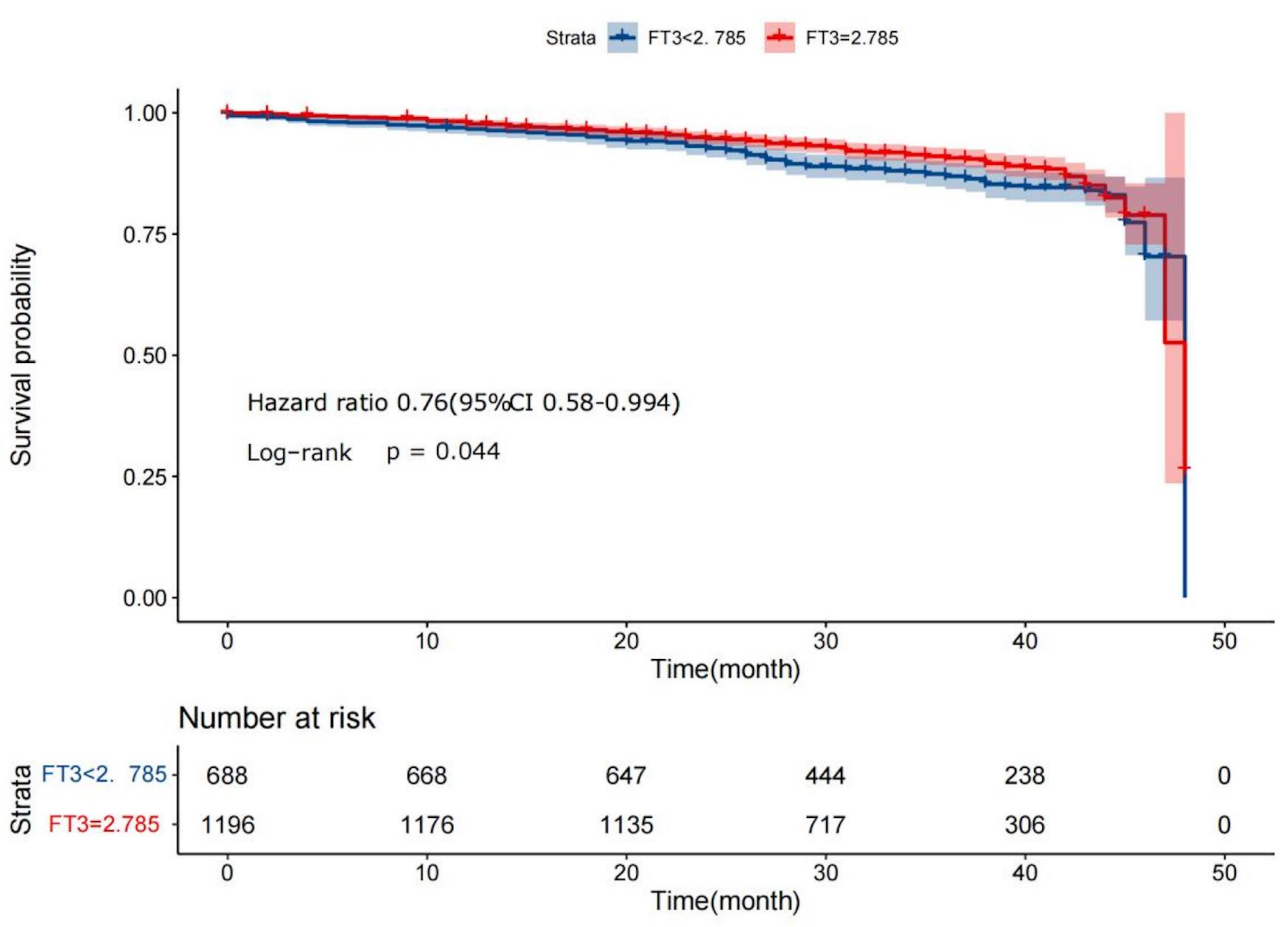
**Kaplan‒Meier survival analysis on FT3 <2.785 group and ≥2.785 group. FT3, free triiodothyronine**.

Patients were divided into the FT3 ≥2.785 group and the FT3 <2.785 group according to the ROC-derived threshold. The findings of the Kaplan‒Meier survival analysis revealed that the incidence of MACEs was considerably lower in the FT3 ≥2.785 group than in the FT3 <2.785 group (Hazard Ratio (HR) = 0.76, 95% CI [0.88–0.994], *p* = 0.044) (Fig. 1).

## 4. Discussion

The main findings of this study were as follows: (1) FT3 was an independent predictor of myocardial infarction, recurrent ISR and TVR in ISR patients after revascularization; and (2) when FT3 was <2.785 pmol/L, the risk ratio of patients with MACE increased significantly.

Angiographic ISR was defined as the loss of ≥50% of the coronary lumen in the area of a previously stented lesion, and stenosis within 5 mm of the edge of the stent as determined by coronary angiography was also defined as ISR [9]. The etiologies of ISR include mechanical and biological factors, where the biological factors include neoplasia, normal hyperplasia, plaque rupture and oxidative stress reactions, among others [10]. In addition, calcified nodules in stents and stent edge effects are also involved in the pathological progression of ISR [10]. It has been shown that drug-eluting stent ISR is primarily characterized by novel intimal atherosclerosis [11]. The results of this study also indicate that although there was no significant difference in low-density lipoprotein cholesterol (LDL-C) levels between the two groups, the average LDL-C level in both groups was higher than 1.8 mmol/L. These findings indicate that LDL-C is an essential factor in the occurrence of ISR after the first percutaneous coronary intervention (PCI).

### 4.1 The Relationship between Thyroid Function and Coronary Atherosclerosis

Thyroxine, including tetraiodothyronine (T4) and triiodothyronine (T3), is an influential endocrine hormone that maintains the normal growth and development of the human body, the function of various organs, and the balance of calcium [12]. T4 is its main storage form and is transformed into biologically active T3 by type I and type II deiodinases [12]. In recent years, it has been shown that T3 not only functions as an endocrine hormone but is also widely involved in the regulation of lipid metabolism [13], endothelial function [14], angiogenesis [15, 16], blood pressure, and myocardial contractility [12], all of which are closely linked to the formation and development of atherosclerosis. In recent years, a number of studies have found a correlation between thyroid hormone and acute coronary syndrome (ACS), and low FT3 is an independent risk factor for coronary artery severity and MACE after PCI [17, 18, 19, 20, 21, 22]. Studies have also explored the relationship between thyroxine and ISR [23]. Canpolat *et al*. [23] reported that elevated plasma FT4 levels were an independent risk factor for ISR after BMS implantation, but few studies have investigated the relationship between FT3 and ISR. In this study, it was found that low FT3 was an independent risk factor for myocardial infarction, recurrent ISR, and TVR in patients with ISR after revascularization, and its mechanism may involve the following aspects.

### 4.2 Effect of Thyroxine on Lipid Metabolism

Patients with hypothyroidism often have abnormal lipid profiles. Tian *et al*. [24] found that TSH activated the cAMP/PKA/CREB signaling pathway by binding to TSH receptors on the surface of the rat liver cell membrane. This directly upregulates the expression of 3-hydroxy-3-methylglutaryl-CoA reductase (HMG-CoA reductase) and promotes cholesterol synthesis. Bakker* et al*. [13] found that T3 can upregulate the expression of LDL-C receptor genes on the surface of the liver cell membrane, increasing the synthesis of LDL-C receptors and thus accelerating the clearance of LDL-C from the circulation. However, Bonde *et al*. [25] observed that proprotein convertase subtilisin/kexin type 9 (PCSK9) levels in hyperthyroid patients were approximately 22% lower than those in the normal population and that FT3 levels were significantly negatively correlated with plasma total cholesterol, very low density lipoprotein cholesterol (VLDL-C), and LDL-C. It was hypothesized that FT3 can reduce plasma LDL-C levels by upregulating the expression of the LDL-C receptor gene and lowering the concentration of PCSK9 in hepatocytes. The event group had considerably lower FT3 levels than the non-MACE group in this study, but there was no significant difference in lipid profiles between the two groups. This result could be related to the diminished effect of FT3 on lipid levels in both groups due to statin use.

### 4.3 Effect of Thyroxine on Vascular Endothelial Cells and Smooth Muscle Function

T3 is also involved in the regulation of vascular endothelial cell function. When Carrillo-Sepúlveda *et al*. [14] studied the effect of thyroxine on the thoracic aorta of rats, it was found that T3 activated nitric oxide (NO) synthase in endothelial cells and smooth muscle cells via the phosphatidylinositol 3-kinase/protein (PI3K/Akt) signaling pathway, resulting in NO production that triggered a rapid diastolic response in the smooth muscle of the vessel. This regulatory function is weakened when T3 levels decrease.

T3 also inhibits vascular calcification. Sato* et al*. [26] reported that T3 affected the expression of genes associated with calcification in rat smooth muscle cells of the aorta. In *in vitro* studies using cultured human coronary smooth muscle cells, physiological concentrations of FT3 (15 pmol/L) increased the mRNA levels of matrix Gla protein (*MGP*), which is considered to be a potent inhibitor of vascular calcification *in vivo*. These results suggest that physiological concentrations of thyroid hormone can directly promote the expression of the *MGP* gene in smooth muscle cells via thyroid hormone nuclear receptors, thereby preventing vascular calcification *in vivo*. Ittermann *et al*. [27] noted that thyroid hormone reduction modifies the smooth muscle structure of the arteries and causes thickening of the vessel walls and decreased compliance, which further aggravates atherosclerosis.

Vascular endothelial cells are damaged by the implantation of coronary stents, and physiological contractions and diastolic function of the vessel walls are inhibited. When the plasma FT3 concentration is reduced, NO secretion by endothelial cells is further suppressed, and the inhibition of vascular calcification is weakened.

### 4.4 T3 is Involved in the Regulation of the Oxidative Stress Response

Oxidative stress plays a crucial role in myocardial ischemia‒reperfusion (I/R) injury. Studies [28, 29] have shown that T3 can increase the activity of superoxide dismutase (SOD) and glutathione peroxidase (GSH-px) in myocardial cells, reduce the production of reactive oxygen species (ROS), and protect against myocardial I/R injury. In the ischemia‒reperfusion injury model, T3 supplementation significantly reduces I/R-induced ROS production and mitochondrial superoxide concentrations, thus acting as a myocardial protective feature.

In addition, the proportion of patients treated with ticagrelor as outpatients was higher in the MACE group than in the non-MACE group in this study. This finding may be due to the fact that follow-up physicians switched to ticagrelor instead of clopidogrel because of MACE events occurs.

### 4.5 Other Factors that may Cause ISR after PCI

​Although there was no significant difference in MACE incidence between diabetic and non-diabetic patients in this study, a number of previous studies have found a higher rate of ISR in diabetic patients after PCI [30, 31, 32, 33, 34]. Study [30] has shown that insulin resistance and elevated cytokine levels are associated with a higher incidence of ISR after PCI even in patients with normal glucose tolerance. Elevated inflammatory markers (interleukin 6; interleukin 1; tumor necrosis factor alpha; C-reactive protein, etc.) and impaired endothelial function can be detected in pre-diabetic patients with coronary heart disease, resulting in abnormally elevated levels of this cytokine after PCI [31]. Overactive inflammatory pathways that cause clotting can also cause restenosis in coronary stents and a poorer prognosis [32, 33]. Currently, it is believed that sodium-glucose cotransporter 2 (SGLT2)-inhibitors can improve clinical outcomes in patients after PCI [34]. However, the relationship between SGLT2-inhibitors and cardiovascular outcomes in PCI patients was not widely recognized at the time this study was initiated 10 years ago and the proportion of diabetics using SGLT2-inhibitors in this study was extremely low. The effect of SGLT2-inhibitors on interventional therapy in ISR-CTO patients with diabetes mellitus was not analyzed.

## 5. Limitations

This study has certain limitations. First, the study was a single-center retrospective cohort study, and most of the patients included in the ISR study had their first interventions completed at different hospitals in China. Therefore, detailed stent information (diameter and length) could not be collected for the patients’ first intervention, making it impossible to complete an effective comparison of relevant stent information. Second, due to the retrospective study design, some indicators related to ISR (such as the matrix metalloproteinase family and secretory frizzled-related proteins) were not included, and the study indicators were limited. Additional, intravascular imaging was also lacking in this study; therefore, some mechanic factors, such as stent underexpansion, could not be assessed. Finally, due to the design of the observational study, it was not possible to investigate whether treatment with thyroxine improved outcomes in ISR patients with reduced FT3 levels. This is expected to be addressed in future prospective clinical studies of such patients.

## 6. Conclusions

Low FT3 is a predictor of myocardial infarction, recurrent ISR and TVR in patients with ISR after revascularization. When the FT3 level is decreased (FT3 <2.785 pmol/L), the risk of postoperative MACE recurrence in ISR patients is significantly increased.

## Data Availability

Datasets used or analyzed during the current study are available from the corresponding author on reasonable request.
